# Innate Anti-microbial and Anti-chemotaxis Properties of Progranulin in an Acute Otitis Media Mouse Model

**DOI:** 10.3389/fimmu.2018.02952

**Published:** 2018-12-14

**Authors:** Zimeng Wang, Qian He, Xinxin Zhang, Yurong Ma, Fangmei Fan, Yilin Dong, Wenchun Xu, Yibing Yin, Yujuan He

**Affiliations:** ^1^Key Laboratory of Diagnostic Medicine, Ministry of Education, Department of Laboratory Medicine, Chongqing Medical University, Chongqing, China; ^2^Department of Laboratory Medicine, Chongqing Traditional Chinese Medicine Hospital, Chongqing, China

**Keywords:** progranulin, *Streptococcus pneumoniae*, otitis media, chemokine (C-C motif) ligand 2, phagocytosis

## Abstract

Acute otitis media (AOM) is one of the most common infectious diseases primarily caused by *Streptococcus pneumoniae* (*S.pn*) among children. Progranulin (PGRN) is a multifunctional growth factor widely expressed in various tissues and cells. Studies have confirmed that PGRN is involved in the development of a variety of inflammatory diseases. We found that the expression of PGRN increased significantly in the middle ear of wild mice with AOM. However, its physiological functions in AOM still remain unknown. To examine the role of PGRN during AOM, we established an acute otitis media model in both C57BL/6 wild mice and PGRN-deficient (PGRN^−/−^) mice via transbullar injection with *S.pn* clinical strain serotype 19F. Interestingly, we observed dual results: on one hand, macrophage recruitment notably increased in PGRN^−/−^ mice compared with WT mice; on the other hand, the overall bacterial clearance was surprisingly dampened in PGRN^−/−^ mice. The enhanced recruitment of macrophages was associated with increased production of chemokine (C-C motif) ligand 2 (CCL2), while the decreased bacterial clearance was associated with impaired endocytosis capacity of macrophages. The scavenging ability of bacteria in PGRN^−/−^ mice was recovered with administration of recombinant PGRN. These results suggested a novel dual role of PGRN in affecting the activities of macrophages.

## Introduction

Acute otitis media (AOM) is the most common pediatric disease around the world. In developed countries, 50–85% of children under 3 years of age suffer from AOM (1). Although AOM has a property of self-limiting, there are approximately 10–20% cases developing into chronic otitis media, which can cause long-term conductive or permanent neurological hearing loss (2). Based on the middle ear lavage fluid (MELF) isolated from AOM patients, clinical analysis revealed that about 50% of AOM cases are caused by *S.pn* (3). It is generally believed that neutrophils play a critical role in the host immune response against Gram-positive bacteria. Upon *S*.*pn* infection, neutrophils are the earliest effector cells recruited to the infected site, no matter in asymptomatic nasopharynx colonization or invasive lung infection (4). Our previous studies (5, 6) confirmed that neutrophils are the predominant effector cells infiltrating the middle-ear cavity during the early stage of AOM. Neutrophils gradually declined from day 3 post-infection, and macrophages were recruited to the middle-ear cavity, suggesting a significant role for macrophages during the late stage of AOM.

PGRN, also known as granulin epithelin precursor (GEP), PC-cell-derived growth factor (PCDGF), proepithelin, and acrogranin, is a 65 kDa secreted glycoprotein that consists of 7.5 granulin domains. PGRN, widely expressed by epithelial cells, immune cells, neurons, microglia, and chondrocytes, plays a critical role in a variety of physiologic and disease processes, including early embryogenesis (7), wound healing (8), cell proliferation (9), cartilage development (10), insulin resistance (11), tumorigenesis (12), and neurodegeneration (13). Recent findings suggested that PGRN acted as an important modulator in inflammatory process, mediating its anti-inflammatory effects by blocking TNF-α binding to its receptors (TNFR1, TNFR2 and DR3) (14). Of interest, the exact function of PGRN might vary depending on the stage and contexts involved in a variety of inflammatory diseases, such as rheumatoid arthritis (15), osteoarthritis (16), inflammatory bowel disease (17), contact dermatitis (18), and psoriasis (19). However, whether PGRN can inhibit the middle ear inflammation and ultimately ameliorate the AOM is still unclear.

In this study, we established an *S.pn* AOM mouse model by transbullar puncture and carefully observed the difference of bacterial load and inflammatory response between WT and PGRN^−/−^ mice, aiming to evaluate the role of PGRN in the development of AOM. Our data indicated high expression of PGRN in the MELF during AOM. Compared with WT mice, PGRN deficiency impaired *S.pn* clearance and led to more severe inflammation. Furthermore, PGRN inhibited tissue inflammation by impeding the recruitment of macrophages regulated by CCL2.

## Materials and Methods

### Bacteria

*Streptococcus pneumonia* clinical isolate 31,693 (serotype 19F) were obtained from the National Center for Medical culture Collections (CMCC, Beijing, China) and grown in casein hydrolysate plus yeast extract (C+Y) medium at 37°C in 5% CO_2_ until mid-log phase (OD_600 =_ 0.4~0.5). After centrifugation, the pneumococcal pellet was washed and resuspended to a density of 1 × 10^7^ CFUs/ml as previously described (19).

### Mice

C57BL/6 mice aged 6 to 8 weeks were obtained from Chongqing Medical University. PGRN^−/−^ mice (B6.129-Tlr2^tmlKir^/JNju) on C57BL/6 background were purchased from the Jackson Laboratory. All mice housed in a specific-pathogen-free environment. The following experiments were done in accordance with the Institutional Animal Care and Use Committee's guidelines at Chongqing Medical University.

### Mouse Model of AOM

The mouse model of AOM was established via transbullar injection followed by the method described previously (20). In brief, mice were anesthetized with an intraperitoneal injection of ketamine hydrochloride (0.020 mg/g of body weight) and xylazine (0.005 mg/g of body weight). Through a ventral midline incision in the neck, the tympanic bulla was exposed bilaterally after blunt dissection. The bony wall of the bulla was fenestrated using a 25-gauge needle and a total of 5 × 10^5^ CFU *S.pn* in about 5 μl was injected slowly into the middle ear cavity with a thinner needle. A mock control cohort of mice was inoculated with an equivalent volume of PBS alone. In some cases, 5 μl PBS containing recombinant murine PGRN (rmPGRN0.4 μg/ml) (R&D Systems, Minneapolis, United States) and 5 × 10^5^ CFU of *S.pn* was injected. After that, the skin incision was sutured. Behavior and weight changes were monitored daily following injection.

### Cell Quantification and Bacterial Load Determination

Mice were sacrificed and intracardially perfused with 10 ml sterile PBS. The tympanic bullae were harvested and spilt into dorsal and ventral halves as described above. The exposed middle ear space was lavaged three times with 40 μl sterile PBS containing 5% fetal calf serum and total MELF (about 120 μl) were collected. Pneumococcal load was quantified by plating 10 μl serial dilutions of MELF onto Columbia sheep blood agar plates and incubated overnight at 37°C in a 5% CO_2_ atmosphere. Another 10 μl MELF was added to 190 μl 1% acetic acid solution for total cell quantification based on standard morphological criteria using a blood cell counting plate. The remaining MELF were centrifuged at 500 *g* for 10 min and supernatants for cytokine measurement were pooled and stored at −70°C until use.

### ELISA

The levels of PGRN were determined by mouse PGRN enzyme-linked immunosorbent assay (ELISA) kit (RayBiotech, Guangzhou, China). Assessment of inflammatory cytokines or chemokines, including tumor necrosis factor α (TNF-α), interleukin 6 (IL-6), interleukin 1β(IL-1β), interferon γ (IFN-γ), interleukin 10 (IL-10), chemokine (C-X-C motif) ligand 1 (CXCL1), interferon gamma-induced protein 10(IP-10) and chemokine (C-C motif) ligand 2 (CCL2) was performed using ELISA kits (Biolegend, San Diego, United States) according to the manufacturer's instructions.

### Flow Cytometry

The MELF of 5 mice from each group at day 1 and day 3 after *S.pn* inoculation were pooled, centrifuged at 500 *g*. The cell pellets were treated with RBC lysis buffer (Biolegend, San Diego, United States) and nonspecific binding was blocked with Mouse BD Fc Block in stain buffer (BD Pharmingen, San Jose, United States). A cocktail of fluorophore-conjugated rat anti-mouse cell surface antibodies included APC-CD45 (BD Pharmingen, San Jose, United States), PE-Cy5-F4/80 (eBioscience, San Diego, United States), FITC-Ly6G (BD Pharmingen, San Jose, United States), PE-CD11b (BD Pharmingen, San Jose, United States), and their isotype antibodies were added for 45 min on the ice. After that, the cells were washed three times and resuspended in stain buffer prior to FACS analysis on a BD FACSCalibur flow cytometer (BD Biosciences, San Jose, United States).

### Histology

Mice were sacrificed under general anesthesia and intracardially perfused with 10 ml 4% paraformaldehyde (PFA) at designed time post inoculation. The tympanic bullae were subsequently dissected, fixed overnight in 4% PFA and decalcified for 4 weeks in 10% EDTA. After that, they were embedded in paraffin, sectioned at 7 μm, stained with hematoxylin and eosin (HE) and digitally recorded with Nikon ECLIPSE 80i.

### Immunohistochemistry

Middle-ear sections were deparaffinized, rehydrated and then incubated in 0.125% trypsin solution to unmask antigens. Endogenous peroxidase was removed by 0.3% H_2_O_2_. The sections were subsequently blocked with 5% BSA for 1 h at room temperature (20–25°C), incubated with rabbit anti-PGRN primary Ab (1:100; R&D Systems, Minneapolis, United States) at 4°C overnight and HRP-labeled goat anti-rabbit secondary Ab (Zhongshanjinqiao, Beijing, China), which was developed by a diaminobenzidine substrate kit for peroxidase and then counterstained with hematoxylin. Immunohistochemical images were obtained using a digital microscope.

### Immunofluorescence

For pneumococcal clearance, MELF were pooled from at least four mice, 100 μl were spun onto slides at 700 rpm for 5 min, desiccation for 15 min. After fixed in 2% PFA for 10 min, the cytospin preparations were blocked with 5% normal donkey serum, and then incubated with rabbit anti-pneumococcal polysaccharide polyclonal antibody (1:1,000; Staten Serum Institute, Denmark) overnight at 4°C. After further washing in PBS, an Alexa Fluor 488-conjugated donkey anti-rabbit IgG (1:800; Jackson ImmunoResearch, United States) was added for 1 h at room temperature. The slides were washed in PBS and counterstained with DAPI (1:1,000; Invitrogen, California, United States). Immunofluorescence images were collected using Nikon ECLIPSE 80i.

### Isolation and Culture of Macrophages and Neutrophils

Macrophages were elicited in mice peritoneal cavity via intraperitoneal injection of 1 ml paroline and then harvested from peritoneal lavage 7 days later. The lavage was centrifuged, and the resulting pellet was resuspended in RBC lysis buffer to remove red blood cells. Subsequently, after washed with ice-cold PBS twice, macrophages were collected by centrifugation at 500 g for 5 min and then cultured in 24-well plates at 1 × 10^5^ cells/ml in DMEM (Gibco, New York, United States) containing 10% fetal bovine serum (FBS) for 4–6 h in a 37°C, 5% CO_2_ atmosphere.

For isolation of neutrophils, the bone marrow cells were flushed from tibia and femur with 1% FBS-RPMI 1640 (Gibco, New York, United States), and then purified by discontinuous Percoll gradient centrifugation with magnetic cell sorting (Miltenyi Biotec, USA). Neutrophils were cultured in 24-well plates at 1 × 10^6^ cells/ml in 10% FBS-RPMI1640 for 2 h in a 37°C, 5% CO_2_ atmosphere.

### Endocytosis Assays

FITC-labeled *S.pn* was prepared by incubation with 0.5 mg/ml FITC (Sigma, Poole, United Kingdom) for 20 min at 37°C. Peritoneal macrophages (1 × 10^5^ cells) and neutrophils (1 × 10^6^ cells) from PGRN^−/−^ and WT mice were pretreated with or without rmPGRN (100 ng/ml, R&D systems, Minneapolis, United States) for 2 h, and then infected with FITC-labeled *S.pn* (at multiplicity of infection, MOI, of 10). After washing steps, cell nuclei were stained with DAPI (Invitrogen, California, United States), followed by visualization using confocal laser scanning microscopy (LSM 510, Zeiss). The ratio of engulfed bacteria (as determined by overlay of green bacteria) was quantified by an independent researcher from 100 counted cells per well and was expressed as percentage of cells that contain bacteria.

### Bacterial Killing Assays

*S.pn* was grown to logarithmic phase in C+Y to OD _600 =_ 0.5 (1 × 10^8^ CFUs/ml). Peritoneal macrophages (1 × 10^5^ cells) and neutrophils (1 × 10^6^ cells) from PGRN^−/−^ and WT mice were pretreated with or without rmPGRN (100 ng/ml) for 2 h, and then infected with *S.pn* (MOI of 100) for 0.5 h for evaluation of bacterial uptake, then immediately, extracellular bacteria were removed by washing with 10 μg/ml Penicillin and 200 μg/ml gentamicin. Neutrophils/macrophages were continued to incubate 1 h for evaluation of intracellular killing (total time = 1.5 h), respectively. Cells were lysed and live intracellular bacteria loads were determined by plating cell lysate on Columbia CNA agar plate. The colonies were counted after overnight incubation at 37°C in a 5% CO_2_ atmosphere. Intracellular killing was calculated as follows: (number of CFUs _t = 0.5h_ −number of CFUs _t = 1.5h_)/ number of CFUs _t = 0.5h_.

### Statistical Analyses

All statistical analysis was performed using GraphPad prism software version 5.01 for Windows (GraphPad, La Jolla, CA, United States). For analysis of CFU data, Mann-Whitney *U*-test was performed. For analysis of all other data, unpaired *t*-test was performed. *P* < 0.05 was considered statistically significant.

## Results

### PGRN Production Was Up-Regulated in Experimental *S.pn* AOM

**Figure 1:**
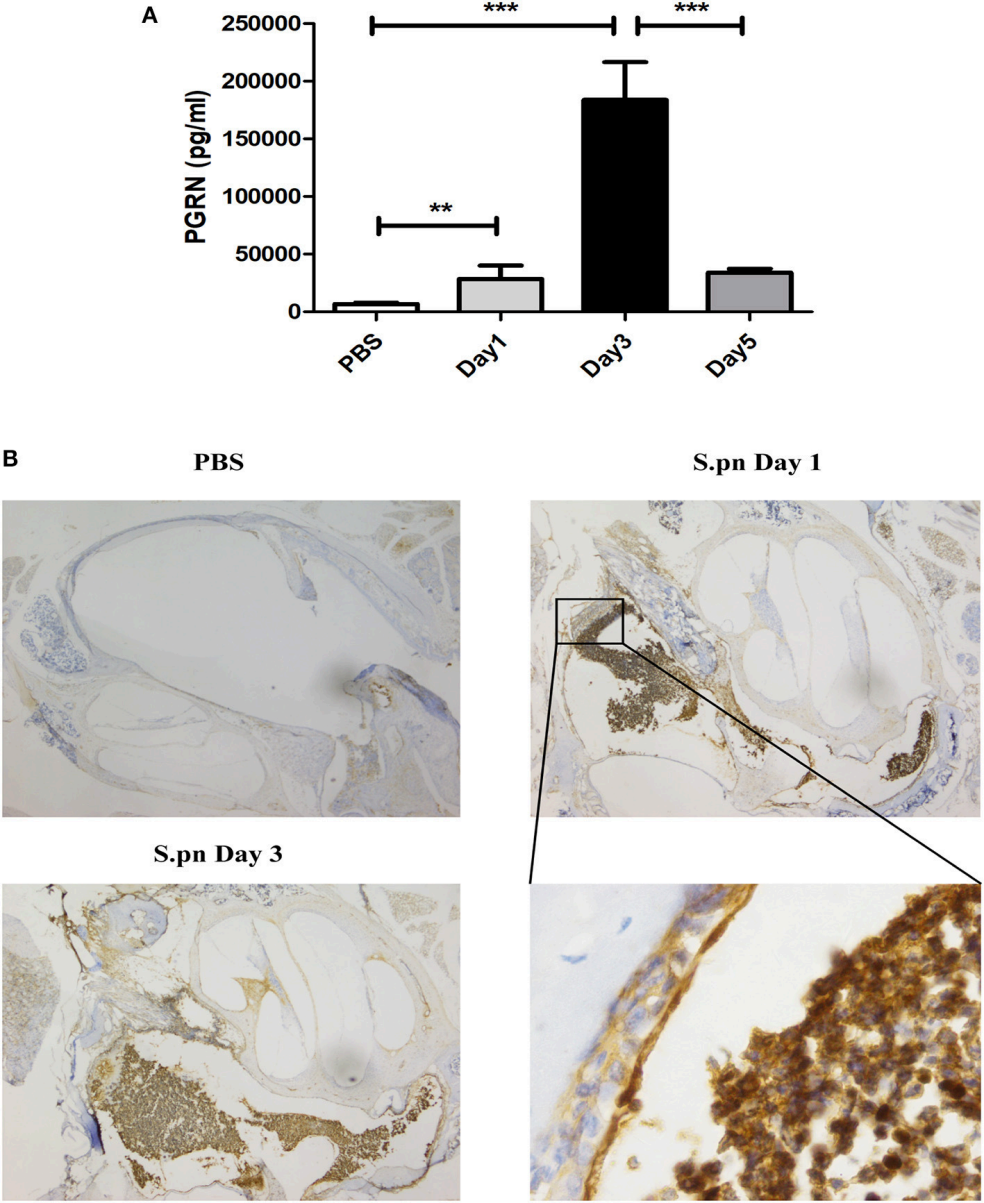
PGRN is significantly induced in response to *S.pn* during AOM. **(A)** PGRN protein levels in the supernatants of MELF were detected by ELISA at designated time points post-infection. **(B)** Immunohistochemical staining of PGRN on middle-ear sections at indicated time points after PBS or *S.pn* inoculation. Control stains were performed with omission of rabbit anti-PGRN primary antibody. Data represent three independent experiments and are presented as mean ± SD (*n* = 5). ^**^P < 0.01; ^***^P < 0.001.

AOM was induced by *S.pn* clinical strain 19F in C57BL/6 mice, and control group mice were inoculated with sterile PBS. Compared with the PBS group, the expression of PGRN in MELF of *S.pn* group was significantly increased. The rising level peaked at day 3 and declined gradually thereafter (Figure 1A). Meanwhile, PGRN was observed to be highly expressed in both middle ear epithelial cells and infiltrated inflammatory cells in AOM mice by immunohistochemical staining (Figure 1B).

### PGRN Deficiency Elevated Local Inflammatory Cell Levels and Inflammatory Responses

**Figure 2:**
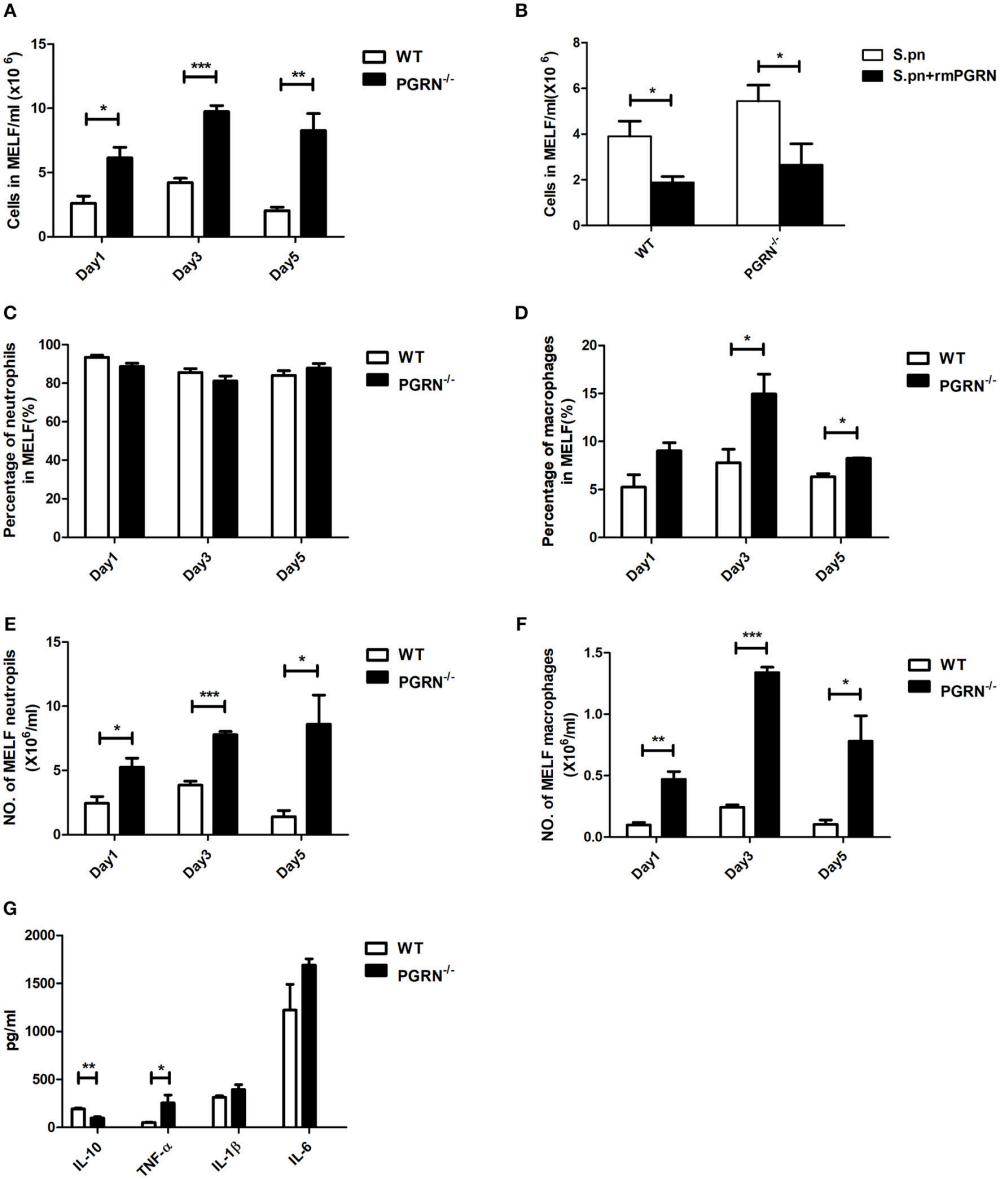
PGRN inhibits inflammatory cell levels and inflammatory responses during AOM. **(A)** The numbers of inflammatory cells in MELF at designated time points post-infection of WT mice and PGRN^−/−^ mice are shown. **(B)** The numbers of inflammatory cells in MELF of WT mice and PGRN^−/−^ mice following transbullar inoculation of *S.pn* or *S.pn* plus rmPGRN at day 3 post-infection are shown. **(C–F)** The ratio of neutrophils (CD11b^+^Ly-6G^+^) and macrophages (CD11b^+^F4/80^+^) was determined, and the absolute number of the cells was calculated. **(G)** Cytokine release in the MELF of WT mice and PGRN^−/−^ mice on day 3 after *S.pn* or *S.pn* plus rmPGRN inoculation. Data represent three independent experiments and are presented as mean ± SD (*n* = 5). ^*^P < 0.05; ^**^P < 0.01; ^***^P < 0.001.

To determine the role of PGRN in AOM, we compared inflammatory cell numbers in MELF between PGRN^−/−^ mice and WT mice at different time points after *S.pn* inoculation. From day 1 post-inoculation, we observed an increase in absolute number of inflammatory cells in PGRN^−/−^ mice vs. WT counterparts. The rising reached its peak at day 3 and lasted till day 5 (Figure 2A). We evaluated the cytotoxicity of rmPGRN before administration of exogenous recombinant murine PGRN. Different concentrations of rmPGRN were applied to neutrophils and macrophages, and then we observed cell viability through trypan blue staining to choose an appropriate dosage of rmPGRN (data not shown). As expected, inoculation with *S.pn* plus rmPGRN (2 ng/ear) reduced the inflammatory cells in WT and PGRN^−/−^ mice (Figure 2B). Similarly, we observed the same results by hematoxylin and eosin (H&E) staining of histologic sections (Supplementary Figures S1, S2). To further clarify PGRN-driven inflammatory cell types, we conducted classified quantification of the inflammatory cells in MELF by flow cytometry. Data showed that on day 1 after *S.pn* inoculation, more than 93% of the MELF cells in WT mice were neutrophils (CD11b^+^Ly-6G^+^ cells), whereas about 85% in PGRN^−/−^ mice. Importantly, the macrophage (CD11b^+^F4/80^+^) percentage in WT mice was about 3%, whereas in PGRN^−/−^ mice it accounted for 7–8%. On day 3, the macrophage percentage in PGRN^−/−^ mice noticeably rose to 14%, while it slightly rose to 5–6% in WT group (Supplementary Figure S3). Compared with WT mice, the percentage and absolute number of macrophages in MELF were both elevated in PGRN^−/−^ mice, but only the absolute number of neutrophils in MELF was elevated in PGRN^−/−^ mice (Figures 2C–F). Likewise, our histopathology results also showed that more macrophages appeared in the middle ear cavity of PGRN^−/−^ mice than those in WT mice on day 3 (Supplementary Figure S4). Taken together, these data suggested a role of PGRN to suppress recruitment of innate inflammatory cells, especially macrophages, in *S.pn*-induced AOM model.

Cytokine profiles were also analyzed at day 3. Compared with WT mice, PGRN^−/−^ mice exhibited high expression of TNF-α, whereas the anti-inflammatory cytokine interleukin-10 (IL-10) secretion was significantly down-regulated (Figure 2G). Besides, the expressions of IL-1β and IL-6 were slightly increased, but with no statistical significance.

### PGRN Deficiency Promoted Macrophage Recruitment Through CCL2 Production

**Figure 3:**
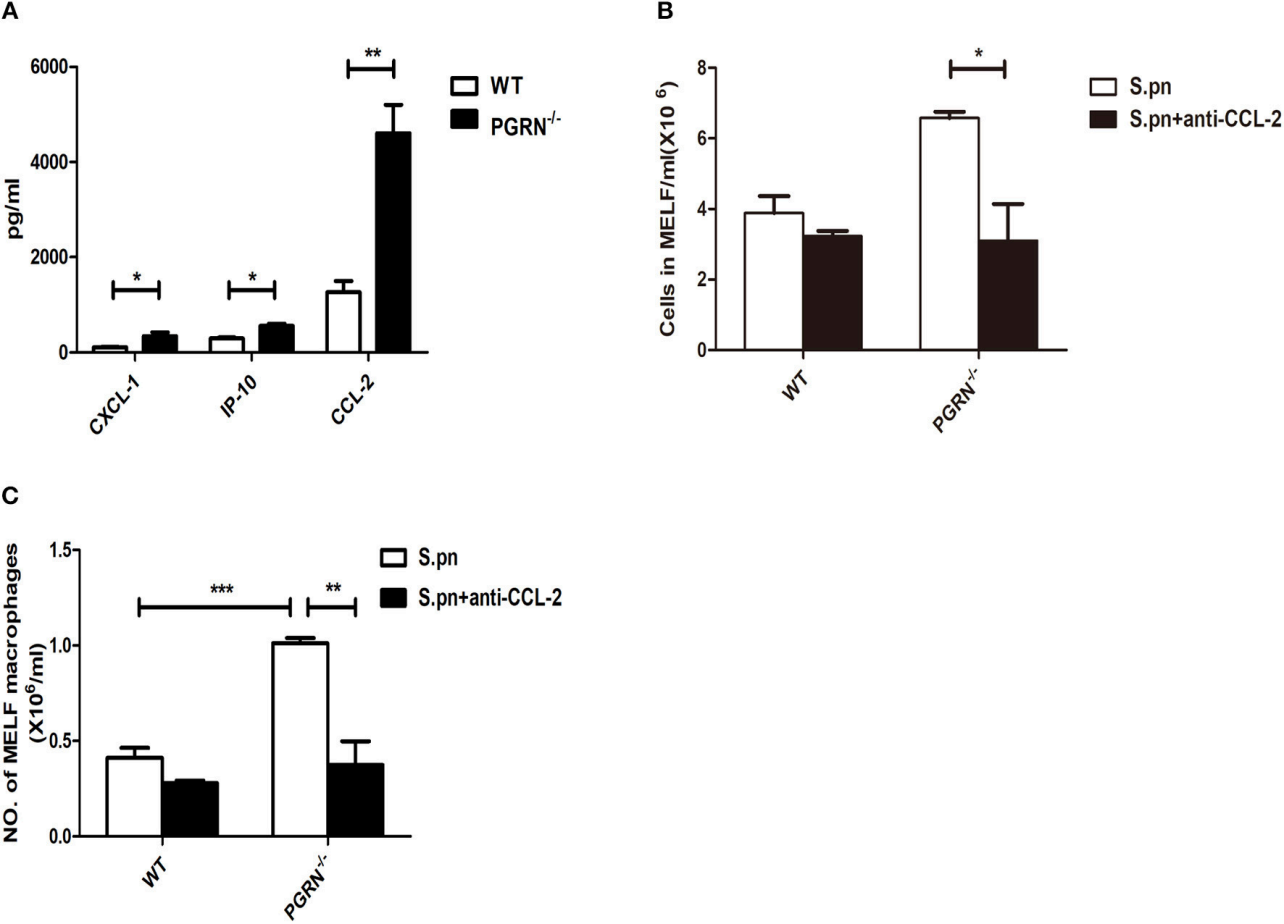
PGRN reduces macrophage recruitment by inhibiting CCL2 expression. **(A)** Chemokine release in the MELF of WT mice and PGRN^−/−^ mice on day 3 after *S.pn* inoculation. The numbers of inflammatory cells **(B)** and macrophages **(C)** in MELF of WT mice and PGRN^−/−^ mice on day 3 after *S.pn* or *S.pn* plus anti-CCL2 inoculation are shown. Data represent three independent experiments and are presented as mean ± SD (*n* = 5). ^*^P < 0.05; ^**^P < 0.01; ^***^P < 0.001.

Since the recruitment of inflammatory cells to the inflammatory site is mediated by chemokines, we then examined the expression of chemokines in the MELF by ELISA. Compared with WT mice, the levels of CXCL1 and CCL2 were both elevated in PGRN^−/−^ mice on day 3. Particularly, the CCL2 level is much higher than CXCL1 (Figure 3A). After administration of anti-CCL2 antibody, a remarkable decrease in the inflammatory cell numbers, especially macrophages, was observed in the MELF of PGRN^−/−^ mice (Figures 3B,C). Likewise, tissue sections of middle ear (ME) results also showed that the inflammatory cells in the middle ear cavity apparently decreased on day 3 after anti-CCL2 inoculation (Supplementary Figure S5). These data evidenced CCL2-driven up-regulation on the recruitment of inflammatory cells.

### PGRN Deficiency Impaired Endocytosis of Macrophages and Neutrophils

**Figure 4:**
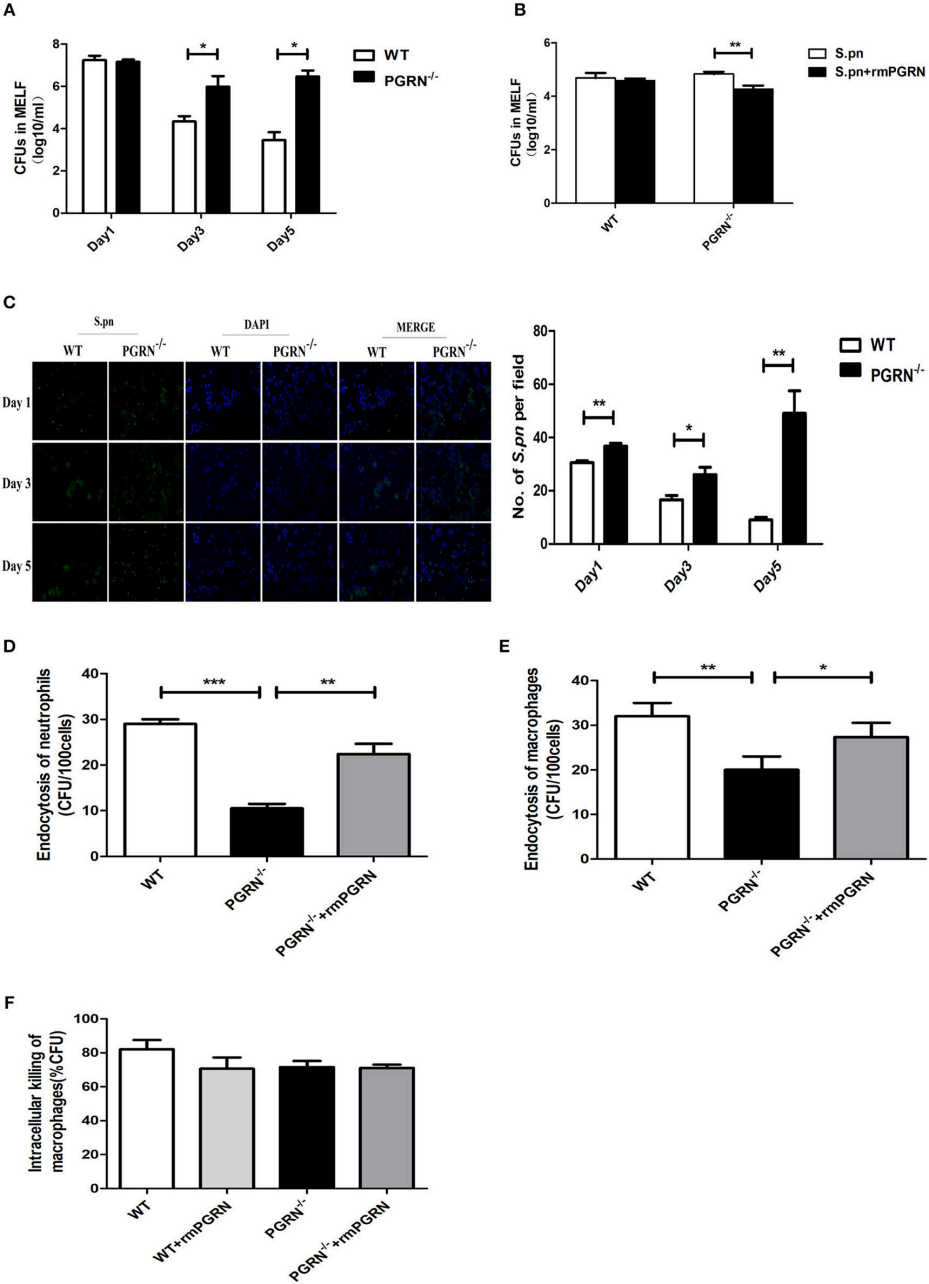
Effects of PGRN on bacterial endocytosis and intracellular killing by macrophages and neutrophils. **(A)** Density of *S.pn* colonization in MELF at designated time points postinfection of WT mice and PGRN^−/−^ mice. **(B)** Density of *S.pn* colonization in MELF at day 3 after *S.pn* or *S.pn* plus rmPGRN inoculation in WT mice or PGRN^−/−^ mice. **(C)** Representative immunofluorescence staining of MELF cytospin preparations from WT mice and PGRN^−/−^ mice at designated time points postinfection. Green represents *S.pn* and blue for cell nucleus. Original magnification, × 10. **(D)** The average number of bacteria engulfed by 100 neutrophils (*n* ≥ 100 cells per condition). **(E)** The average number of bacteria engulfed by 100 macrophages (*n* ≥ 100 cells per condition). **(F)** The intracellular killing ratio of macrophages from WT mice or PGRN^−/−^ mice. Data represent three independent experiments and are presented as mean ± SD (*n* = 5). ^*^P < 0.05; ^**^P < 0.01; ^***^P < 0.001.

To investigate the potential role of PGRN in AOM, we examined bacterial load of MELF in PGRN^−/−^ and WT mice. It was worth noting that the *S.pn* load in PGRN^−/−^ mice was significantly higher than WT mice on day 3, 5 (Figure 4A). Besides, the attenuated bacterial clearance could be recovered by administration of rmPGRN (Figure 4B). Additionally, immunofluorescence staining of the MELF cytospin specimens showed that a large amount of intact pneumococci was detected in the middle ear cavity of PGRN^−/−^ mice on day 5, while only a small amount of pneumococcal debris in WT mice (Figure 4C). Based on our data, we hypothesize that due to PGRN deficiency, the overall bactericidal capacity of inflammatory cells was impaired. It is well-known that phagocytosis of phagocytes is composed of three distinct steps: recognition/binding, internalization/endocytosis and digestion/intracellular killing. We further examined which step was basically modulated by PGRN *in vitro*. The endocytosis activity of PGRN^−/−^ macrophages/neutrophils was suppressed (Figures 4D,E), while the intracellular killing activity was not affected (Figure 4F). The extent of intracellular killing activity of neutrophils is similar to that of macrophages (data not shown). Moreover, we found that pre-incubation with rmPGRN augmented the endocytosis of live *S.pn* by macrophages and neutrophils (Figures 4D,E). In summary, these results demonstrated that PGRN deficiency impaired the overall bacterial clearance by attenuating phagocytosis of macrophages and neutrophils.

## Discussion

Streptococcus pneumoniae is one of the most common pathogens causing a variety of respiratory infectious diseases, such as otitis media, pneumonia, bacteremia, and meningitis. *S.pn* includes at least 97 identified serotypes, and 19F is the prevailing human pathogen from the middle ear of otitis media patients (21). So far, the mechanism of the host immune response against *S.pn* AOM remains largely unknown. To explore the underlying mechanism, we developed a model of AOM in C57BL/6 mice using *S. pn* clinical strain 19F. Infection with this Gram-positive organism characteristically led to hyperplasia of the ME mucosa and leukocyte infiltration in the ME cavity (20). In this study, we demonstrated that PGRN level was elevated in the course of experimental AOM and PGRN mainly inhibited the recruitment of macrophages in the middle ear cavity by suppressing CCL2 expression. Furthermore, PGRN mediated host defense against *S.pn* by influencing endocytic activity of macrophages and neutrophils.

Recently, PGRN is widely investigated as an important modulator of inflammatory process. As a pleiotropic cytokine expressed by various tissues and cells, PGRN can exert trophic and anti-inflammatory activity. During last decade, many studies reported high expression of PGRN in various inflammatory diseases including arthritis (15, 16), inflammatory bowel diseases (17), contact dermatitis (18), psoriasis (19), atherosclerosis (22), systemic lupus erythematosus (23), and type 2 diabetes (24). It has been confirmed that PGRN is a physiological endogenous ligand of TNF receptors (TNFR). PGRN blocks TNF-α-mediated signaling pathways by competing with TNF-αbinding to TNFR1/2, and then suppresses inflammatory response. Tang W. and colleagues confirmed that PGRN prevented inflammation by inhibiting TNFα-activated signaling in multiple arthritis mouse models (25). Yan W. and colleagues also reported that PGRN augmented IL-10 production by LPS-activated macrophages in a TNFR-dependent manner in mouse models of polymicrobial sepsis and endotoxinemia (26). Recently, Ma Y. and colleagues documented that PGRN reduced the release of pro-inflammatory cytokines (IL-1β, TNF-α, and IL-6) in activated microglia during LPS-induced immune stress (27). Our data also showed that the expression of PGRN in the MELF was significantly increased during AOM. Compared with WT mice, PGRN^−/−^ mice exhibited high expression of TNF-α, whereas IL-10 secretion was significantly down-regulated.

Several chemotactic proteins have been confirmed to be implicated in the recruitment of macrophages to sites of inflammation and infection, including CCL2, which is also known as monocyte inflammatory protein 1 (MCP-1). In previous studies, PGRN has emerged as a potential chemotactic molecule regulating macrophage recruitment to control innate immunity and inflammation (28). Byung-Soo Youn and colleagues proposed that the extent of PGRN-mediated chemotaxis is similar to that of MCP-1 (29). He Z et al. indicated that PGRN can increase the accumulation of neutrophils and macrophages in the cutaneous wound (30). Fiona Pickford et al. also demonstrated that PGRN acted as a chemoattractant in brain to recruit or activate microglia (31). These studies suggested that PGRN can function as a chemotactic protein for myeloid-origin cell types. However, many opposite findings emerged recently. In LPS-induced acute lung injury, administration of PGRN effectively decreased the levels of chemokines including CXCL2, CCL2, and KC, and subsequently reduced the total cells including neutrophils in BAL fluid (32). Yusuke Egashira found that r-PGRN treatment suppressed neutrophil recruitment into the ischemia-reperfusion brain (33). Hwang HJ found that PGRN significantly reduced the LPS-mediated expression of MCP-1 in human umbilical vein endothelial cells (HUVECs) (34). In a rat model of Subarachnoid hemorrhage, the recruitment of neutrophils decreased after r-PGRN treatment (35). In a mouse model of renal ischemia/reperfusion injury, PGRN deficiency increased tubulointerstitial neutrophil and macrophage infiltration, which can be attenuated by rPGRN pretreatment (36). The same effect was found in a mouse model of LPS-induced acute kidney injury (37). However, in the mouse models of traumatic brain injury and muscle injury, researchers found that PGRN deficiency did not affect the number of microglia/macrophages (38, 39). In all, the multiple functions of PGRN might be mediated by binding with different partners in a cell-specific or disease-specific manner. In our study, we observed the increasing inflammatory cells (neutrophils and macrophages) in the MELF of PGRN^−/−^ mice vs. WT counterparts during AOM, especially macrophages. The recruitment kinetics of inflammatory cells links to the levels of chemokines (CXCL1 and CCL2). Our results suggested that PGRN plays an anti-chemotaxis role in AOM.

Macrophages are existed in all tissues and cavities, and can play a crucial role in host defense during infection. Specifically, macrophages can eliminate invading pathogenic bacteria in direct or indirect ways through a variety of innate and adaptive immune responses (40). Michael J. Ungurs found that infection of NHBE cells with live Haemophilus influenzae significantly increased PGRN secretion and negatively associated with bacterial colonization in clinically stable COPD (41). The role of endogenous PGRN during bacterial infection was previously studied using PGRN gene knockout mice infected with Listeria monocytogenes, which showed reduced macrophage numbers in infected spleens, resulting in impairing the ability of bacterial clearance compared with wild-type mice (42). S. Sakura Minami et al. investigated the role of microglia-derived PGRN by selectively abrogating PGRN in myeloid cells and found that PGRN enhanced microglia-mediated phagocytosis (43). Hiromi Murase also found that PGRN can increase phagocytosis of the shed photoreceptor outer segments (POS) in retinal pigment epithelial (44). However, the effects of PGRN on macrophage function and host defense in AOM had not been studied. Intriguingly, in spite of the growing numbers of macrophages and neutrophils, the clearance of *S.pn* was delayed in the MELF of PGRN^−/−^ mice vs. WT mice on day 3, 5. Besides, the attenuated bacterial clearance could be recovered by administration of rmPGRN. Considering phagocytosis is a complex process comprising three distinct steps: recognition/binding, internalization/endocytosis and digestion/intracellular killing, we further measured the endocytosis and intracellular killing activities of macrophages/neutrophils, respectively. Our results confirmed that PGRN deficiency impeded the endocytosis activity of macrophages/neutrophils, but had no effect on intracellular killing activity, resulting in impairing bacterial clearance in PGRN^−/−^ mice compared with WT mice. Consistently, Fiona Pickford also found that PGRN can activate microglia and increase endocytosis of extracellular peptides such as amyloid β.

There are a few important limitations to this study. Firstly, we cannot collect the MELF of AOM patients in this study, and thus the diagnostic and prognostic value of PGRN cannot be evaluated in a clinical trial. Secondly, it remains to be determined whether the effects of PGRN deficiency in *S.pn* 19F-induced AOM also can be applied to other *S.pn* serotype–induced and NTHi –induced AOM. Furthermore, the receptor(s) of PGRN and the mechanisms that PGRN regulates CCL2 production in AOM remain unknown. Recently, Hidetoshi Sugihara reported that PGRN deficiency led to an increase of M2 macrophages in regenerating muscle, and prolonged the persistence of macrophages (possibly M2 type), suggesting that M1 and M2 macrophage populations may vary under different health conditions and the outcome of bacterial infection is contingent on dominant macrophage microenvironments (45). In the future research, the subtype change of macrophages and neutrophils will be noteworthy.

In summary, PGRN acts as a protective mediator in AOM. We have demonstrated that PGRN exhibited anti-microbial and anti-chemotaxis properties using an *in vivo* AOM model and *in vitro* macrophage/neutrophil infection models. We hypothesize that PGRN deficiency attenuated the phagocytosis capacities of macrophages and neutrophils, leading to more macrophages and neutrophils recruitment to inflammatory site to compensate the weakened phagocytosis capacities. Further studies are under way aiming at understanding the molecular mechanisms during the regulation of PGRN on macrophages/neutrophils, which are valuable to assess the therapeutic potential of rPGRN.

## Data Availability Statement

The raw data supporting the conclusions of this manuscript will be made available by the authors, without undue reservation, to any qualified researcher.

## Author Contributions

ZW and YH conceived of and designed the research. ZW, QH, XZ, YM, FF, and YD performed the experiments and analyzed the data. ZW and YH wrote the manuscript. WX, YY, and YH interpreted the data and corrected the manuscript.

### Conflict of Interest Statement

The authors declare that the research was conducted in the absence of any commercial or financial relationships that could be construed as a potential conflict of interest.
