# Supplementary material for: Innate Anti-microbial and Anti-chemotaxis Properties of Progranulin in an Acute Otitis Media Mouse Model
Source: Front Immunol. 2018 Dec 14;9:2952. doi: 10.3389/fimmu.2018.02952 (PMC6302024; doi:10.3389/fimmu.2018.02952)
Supplement: Supplementary file 1 [file Data_Sheet_1.docx]

Supplementary Material

**Evidence of the Innate [Anti-microbial](javascript:;) and Anti-chemotaxis Properties of Progranulin in a Mouse Model of Acute Otitis Media**

**Zimeng Wang , Qian He, Xinxin Zhang, Yurong Ma, Fangmei Fan, Yilin Dong, Wenchun Xu, Yibing Yin, Yujuan He***

*** Correspondence:** Corresponding Author: 100951@cqmu.edu.cn

## Supplementary Figures

**
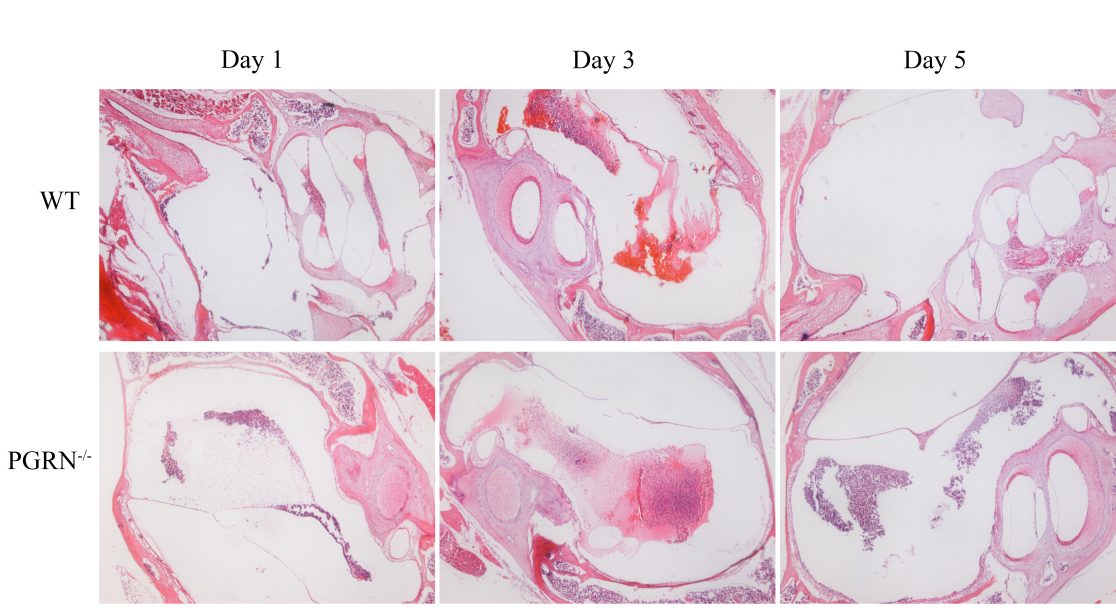
**

**Supplementary Figure 1.** Sections of the middle ear were stained with H&E. Original magnification, ×4.

**
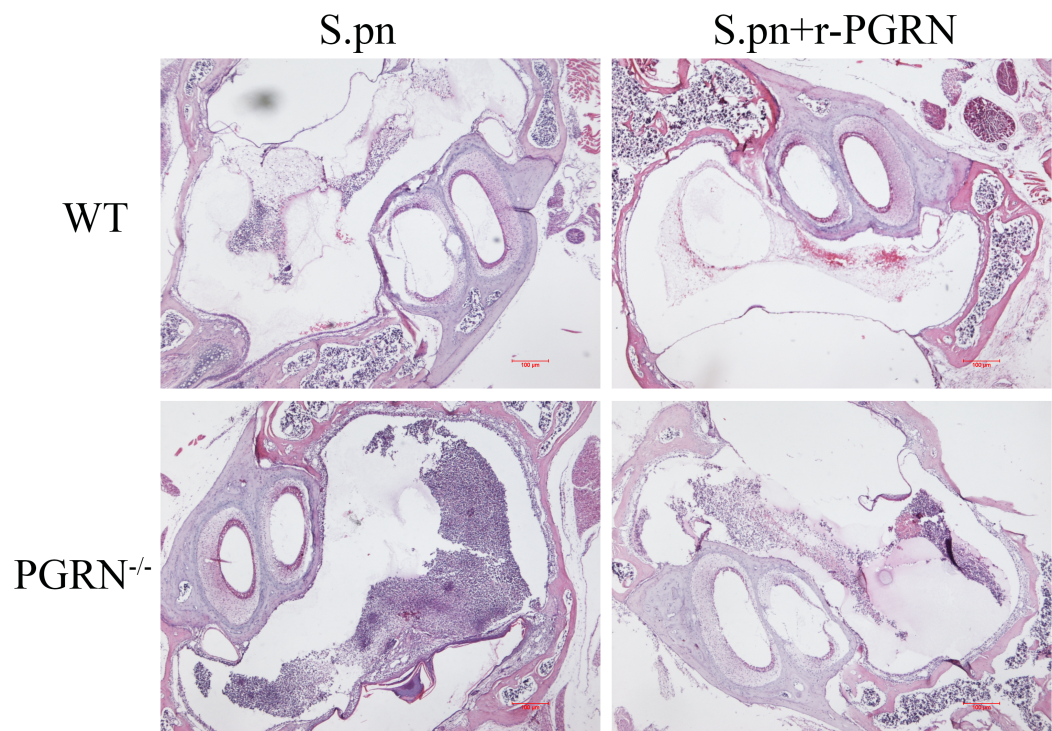
**

**Supplementary Figure 2.** Sections of the middle ear were stained with H&E. Original magnification, ×4.

**
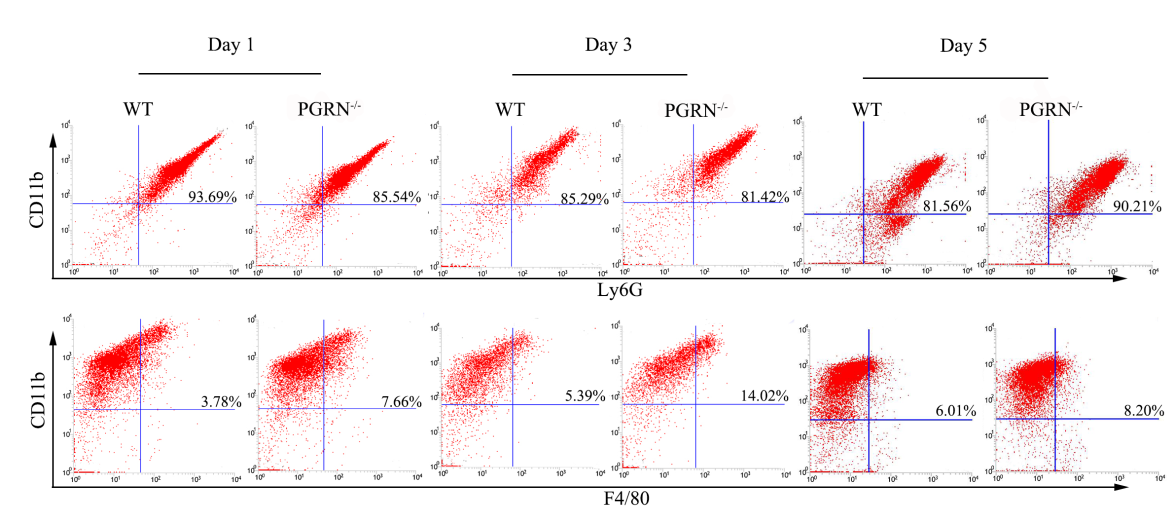
**

**Supplementary Figure 3.** The cells in MELF at designated time points post-infection of WT mice and PGRN^-/-^ mice were stained with monoclonal antibodies against neutrophils and macrophages surface molecules and analyzed by flow cytometry.

**
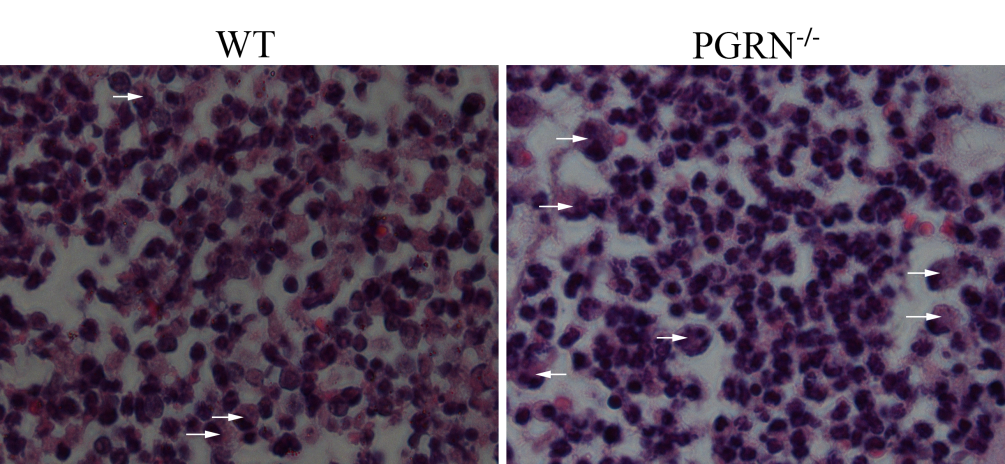
**

**Supplementary Figure 4.** Representative middle ear histopathology at 3 days after S.pn inoculation as shown by H&E staining. Original magnification, ×100. Arrows indicate macrophages.

**
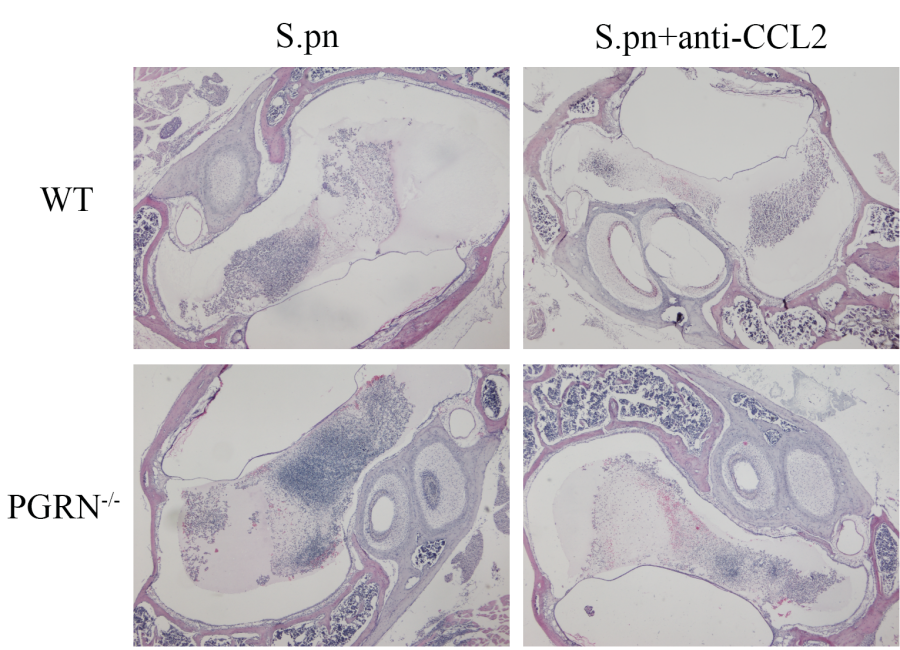
**

**Supplementary Figure 5.** Representative middle ear histopathology at 3 days after S.pn inoculation as shown by H&E staining. Original magnification, ×4.
